# Supplementary material for: Comparative multiomics analysis of cell physiological state after culture in a basket bioreactor
Source: Sci Rep. 2022 Nov 23;12:20161. doi: 10.1038/s41598-022-24687-4 (PMC9686226; doi:10.1038/s41598-022-24687-4)
Supplement: Supplementary file 1 — Supplementary Information 1. [file 41598_2022_24687_MOESM1_ESM.zip › raw data/Proteomic raw data/6.KEGGenrichment/EG.vs.CG/UP/pathway/src/04141.html]

map04141


- K11519, K13989

- Up regulated proteins


A0A0D9RFU7(Description:Derlin;Gene:DERL3;log2FC:1.0356)

- K11519, K13989

- Up regulated proteins


A0A0D9RFU7(Description:Derlin;Gene:DERL3;log2FC:1.0356)

- K09502, K09503, K09507, K09508, K09518, K09525

- Up regulated proteins


A0A0D9R887(Description:DnaJ heat shock protein family (Hsp40) member B12;Gene:DNAJB12;log2FC:1.4208)

- K09502, K09503, K09507, K09508, K09518, K09525

- Up regulated proteins


A0A0D9R887(Description:DnaJ heat shock protein family (Hsp40) member B12;Gene:DNAJB12;log2FC:1.4208)

Close
